# English as She Is Spoke

**Published:** 1885-04

**Authors:** Geo. E. Blackham


### ENGLISH AS SHE IS SPOKE.

GEO. E. BLACKHAM, M. D.

Some time ago quite a little sensation was caused by the publication of a volume with the queer title which heads this article. The book was a sort of hand book for teaching Portugese the practical use of our language, and it was funny, very funny—because of the English given therein—the like of which was never heard on land or sea.

But one could compile a book quite as funny from the actual usage of people who from their social position *ought* to speak good English and who do in fact *write* good English. The written language suffers but little, but the spoken language seems to be undergoing a sort of disintegration, losing an element here and an element there—a sort of a retrograde metamorphosis which threatens to reduce it to the level of some of the savage tongues which are scarcely articulate. Of course we are still very far from such a catastrophe and yet, so rapid has been the disintegration in some localities, that the danger seems painfully real. And the curious thing about it is, that education seems powerless to prevent it.

Take New York city for instance, the commercial and intellectual metropolis of this Nation. Its free school system is its pride and its boast, and yet not one in ten of the graduates of that system with whom I have been acquainted, and I have known many, can give the proper value to the sounds ordinarily represented by the letter "r," or pronounce the aspirate following "w." For instance the word there is pronounced "theyah," the word for "fau," which becomes "witch," what "wat," and so on. I have given the New York pronounciation as nearly as possible, but I find it impossible to render it exactly by any combination of letters in our ordinary alphabet. The strong sound of the "sh" too has become emasculated and weakened into a sort of compromise between "sh" and "zh," the word Asia becoming "Azhia," etc., etc.

It is not only in pronounciation that this loss of differentiation is seen, or rather heard, but in choice of words also, there is a strong tendency to make one word, usually some slang phrase, do duty for many. One evening I heard a young gentleman, a college graduate, use the same phrase to express his admiration of a famous prima donna, and of a famous athlete, and his detestation of a notorious scoundrel; he said "Oh, she's a corker," or as he pronounced it, "Oh, she's a cawca." This appeared to be the utmost he could say to express his delight at Patti's singing, and the same formula, varying the pronoun only, answered to express his opinion of the athlete and the thief. So deep seated is the malady that many of these people have not only lost the power of pronouncing properly, but fail to recognize the sounds when accurately pronounced. A friend of mine, an unusually intelligent young man, and a fellow student of phonography, was one day discussing with me the question of sound analysis, (a most important question to the practical writer of phonetic short-hand,) when I called his attention to his own inability to sound the "h" after "w." He replied: "Wy no; I have no difficulty in sounding the 'h.' I always say 'Wy' and 'wat' same as *you* do —but I have often noticed that you woll the ahs." The young man was tone-deaf and failed to recognize the difference between "wat" and "what," just as a color-blind persons might fail to recognize the difference between red and brown.

The Philadelphia use of "me" for "my" and the New England dislocation of the sound of "r" to the end of words properly ending with a vowel sound are regretable vulgarisms but are less important both from the relatively provincial character of the localities to which they are chiefly confined and because they represent a *misuse* of rather than an actual *loss* of sound elements.

The tendency in the West is rather towards surplusage while that of the East is towards loss. The Western man has half a dozen phrases for a single idea while his eastern compeer is satisfied with one phrase for half a dozen ideas. The Western slang too, is more picturesque and expressive as a rule. My New York friend who spoke so enthusiastically of Mr. Sullivan as "cawca" conveyed to the uninitiated hearer no very definite idea, while no special local knowledge was needed to understand that the person described by a western friend of mine as "half hoss half alligator" would be likely to prove a formidable antagonist in a hand to hand encounter. Another sound that is falling into disuse is that ordinarily represented by "ng" and that represented by "n" is made to do extra duty. We hear of "comin' and goin' and bein'" instead of coming and going and being, &c. So that out of about forty sound elements in all we have three, or more than seven per cent., already placed upon the retired list by a large and influential portion of our citizens.

At this rate, how long would it take to place them all in retirement and reduce our spoken language to a series of inarticulate grunts? The thing that saves us from the probability of such a catastrophe is the wealth of our written and printed language which keeps before us a sort of standard to which we can from time to time return.

There are of course several morals to the sad story, and the first is that it were well if our schools could devote more time to "English as she is, or rather *should be spoke*" even if a portion of the necessary time were filched from Greek or Latin or French or German.

Another moral is, that "We should make haste slowly in the matter of phonetic spelling reform." Of course it would simplify matters if our ordinary spelling were simply placing on paper the characters representing the component sounds of each word; but before we can adopt any such system, we must agree upon some standard of pronounciation, we must teach the Manhattan Island ear to recognize and the Manhattan Island lips to pronounce the aspirate after "w," or *we* must agree to leave it out. Till we can agree upon a common standard pronounciation, we can have no common standard phonetic spelling. Let us learn to talk straight first, and, after that, perhaps, the simplified and corrected orthography may be added unto us.

Meantime, let us cherish and preserve our grand old mother tongue, let our schools teach "English as it should be spoken", and our colleges give it equal rank with the ghosts of dead languages.

Let us endeavor to make our children love it and honor it, and speak it correctly; and above all, let *us* try to speak it correctly ourselves, remembering always, that practice, and practice only, can give us this power, and that we can no more acquire a command of good English by the study of Grammar than a man can become an athlete by the study of anatomy.

